# Association of serum vitamin D status with gestational diabetes mellitus and other laboratory parameters in early pregnant women

**DOI:** 10.1186/s12884-022-04725-9

**Published:** 2022-05-11

**Authors:** Caihong Luo, Zhiju Li, Yunya Lu, Fang Wei, Dongmei Suo, Shiyan Lan, Zhengyuan Ren, Runchang Jiang, Fang Huang, Aiyue Chen, Liejun Jiang, Huayi Huang, Xiaoling Guo

**Affiliations:** 1 Department of Obstetrics, Southern Medical University Affiliated Maternal & Children’s Hospital of Foshan, No. 11 Renminxi Road, Foshan, Guangdong 528000 China; 2 Department of Epidemiology, School of Public Health, Southern Medical University, No. 1838 North Guangzhou Avenue, Guangzhou, 510515 China; 3 Department of Information Technology, Southern Medical University Affiliated Maternal & Children’s Hospital of Foshan, No. 11 Renminxi Road, Foshan, Guangdong 528000 China; 4 Department of Laboratory Medicine, Southern Medical University Affiliated Maternal & Children’s Hospital of Foshan, No. 11 Renminxi Road, Foshan, Guangdong 528000 China; 5 Department of Laboratory Medicine, The People’s Hospital of Guangxi Zhuang Autonomous Region, 6 Taoyuan Road, Nanning, Guangxi 530021 China; 6 School of Medical Laboratory, Youjiang Medical University for Nationalities, No. 98 Chengxiang Road, Baise, Guangxi 533000 China; 7 Mindray North America, 800 MacArthur Boulevard, Mahwah, New Jersey 07430 USA; 8 Department of Surgical Oncology, Roswell Park Comprehensive Cancer Center, Elm and Carlton Streets, Buffalo, New York 14263 USA

**Keywords:** 25-hydroxy vitamin D (25(OH)D), Gestational diabetes mellitus (GDM), Alkaline phosphatase, Pre-albumin, Thrombin time

## Abstract

**Background:**

The association between serum 25-hydroxy vitamin D (25(OH)D) status and gestational diabetes mellitus (GDM) gained attention in recent years, however the conclusion is still controversial due to many interfering factors, such as region of living, environment, lifestyle, and food supplements. Other metabolites (laboratory parameters) are also important in reflecting gestational states. This study aimed to investigate the association of serum 25(OH)D status in early pregnancy with GDM and other laboratory parameters in pregnant women.

**Methods:**

A total of 1516 pregnant women whose blood glucose were normal before pregnancy in the city of Foshan in Guangdong, China were enrolled in this study. GDM was diagnosed between 24 to 28 weeks of pregnancy following the guidelines from the American Diabetes Association. Maternal serum 25(OH)D and other laboratory parameters—including hematology, coagulation, chemistry, and bone density—were measured utilizing various analytical methods in clinical laboratory at gestational weeks 11 to 14.

**Results:**

The average 25(OH)D concentration was 59.1 ± 12.6 nmol/L. None of the study subjects had 25(OH)D < 25 nmol/L; 434 (28.6%) women had 25(OH)D deficiency (< 50 nmol/L), 882 women (58.2%) had 25(OH)D insufficiency (50–74 mmol/L) and 200 women (13.2%) had 25(OH)D sufficiency (≥ 75 nmol/L). There were 264 (17.4%) women diagnosed with GDM. There was not, however, an association between serum 25(OH)D in early pregnancy and GDM. Interestingly, women with more parity and high serum alkaline phosphatase levels had higher serum 25(OH)D levels. There was a possible positive association between serum 25(OH)D and pre-albumin, and a possible negative association between serum 25(OH)D, creatinine, and thrombin time. This study did not find an association between serum 25(OH)D and bone density.

**Conclusions:**

There were no associations between maternal serum 25(OH)D concentration in early pregnancy and the risk of GDM or bone density. There were, however, correlations between serum 25(OH)D and parity, seasoning at sampling, serum alkaline phosphatase, creatinine, pre-albumin, and coagulation factor thrombin time, which need further study to explain their pathophysiology and clinical significance.

## Background

Vitamin D is a fat-soluble vitamin, which plays an important role in bone mineralization, calcium and phosphorus absorption, parathyroid status, and immune system functionality [1]. In the human body, vitamin D from sunlight and food is hydroxylated to 25-hydro vitamin D (25(OH)D) in the liver and is subsequently activated with a second hydroxylation to 1,25-dihydroxy vitamin D in the kidney [2].

Studies have found that during pregnancy calcitriol increases in early gestation to meet a mother’s needs, and maternal serum 25(OH)D crosses into the placenta for use in fetus development [3]. Serum 25(OH)D below 50 nmol/L is defined as vitamin D deficiency, while it is defined as insufficiency if the level of 25(OH)D is in between 52–74 nmol/L, only when the level reaches 75 nmol/L or higher is deemed sufficient [4–8]. Insufficient vitamin D levels may cause some adverse pregnancy complications, including polycystic ovary syndrome, gestational diabetes mellitus (GDM), pre-eclampsia, infertility, endometriosis, and cancers [9–12]. Studies on the association between vitamin D status and pregnancy complications are still controversial because serum vitamin D level is affected by many factors, such as climate, region of living, life style and diet, physical exercise, and food supplements that pregnant women may have consumed during pregnancy. A meta-analysis of 29 observational studies indicated that vitamin D deficiency was related to increased risk of gestational diabetes [13]. Nevertheless, Pérez-López et al. found that there was no association between vitamin D supplementation and the risk of GDM in a systematic review and meta-analysis of randomized controlled trials [14]. Thus, in this study, we wanted to see whether there is an association between serum 25(OH)D level and adverse pregnancy effects, including GDM, in pregnant women living in the Foshan area of Guangdong Province in China.

In addition, laboratory parameters and metabolites may indicate certain clinical complications of the pregnant women and thus guiding the clinical management. The greatest magnitude increase in multiple micronutrient supplements use occurred in women of parity ≥ 2 [15]. Li et al. reported that vitamin D played a role in promoting alkaline phosphatase (ALP) activity [16]. While a systematic review and meta-analysis of interventional studies showed association between serum 25(OH)D and bone density [17]. In addition, vitamin D supplementation might have contributed to liver and kidney fuction [18]. Interestingly, Bouillon [19] mentioned that vitamin D might affect blood coagulation. Therefore, we also had an interest in understanding whether parity, bone density, clinical chemistry and hematology parameters such as serum ALP, creatinine (Cre), pre-albumin (Pre-Alb), coagulation factors including thrombin time (TT), activated partial thromboplastin time (aPTT), D-dimer, and fibrinogen (Fib) level have anything to do with serum vitamin D, GDM, and pregnant adverse effects. Following these concepts, the routine clinical laboratory parameters as mentioned above were screened and analyzed as an important part of our observation in this study.

Our study followed the policies regarding the management and medical practice on pregnant women and newborns by the Chinese Medical Association and Chinese government, which require all pregnant women be closely monitored during pregnancy. We therefore proposed this study to offer a more detailed observation of the metabolic criteria, including the association between maternal serum 25(OH)D levels in early pregnancy, GDM, and other laboratory parameters in pregnant women. We hoped to better understand metabolic events and their potential effects on fetus development, as well as their potential diagnostic and prognostic applications.

## Patients and methods

### Patient enrollment and ethics approval

This was a prospective cohort study for a grant supported by the Foshan Science and Technology Bureau Project (2018AB000251) of Guangdong, China. During the study, a total of 241,651 pregnant women who visited our hospital for scheduled check-ups during the prenatal period at Foshan Women and Children’s Hospital of Southern Medical University, a specialized tier-3, grade-A hospital in South China, were examined. Of them, 12,536 women were giving birth, of which after excluding those with medical conditions such as diabetes and hypertension as described below, and also on voluntary enrollment basis, a total of 1,516 women, ages 18 to 46, were recruited from August 1, 2018 to August 31, 2019. The relevant information of those early pregnant women who met the criteria of the study was collected and serum 25 (OH) D and other laboratory testing were performed during early pregnancy. Pregnant women continued regular antenatal examination and follow-up until the termination of pregnancy. Women who had pregestational diabetes mellitus (PGDM), malignant diseases or missing OGTT data, or pregestational hypertension were excluded from the study. Exclusion criteria included pre-existing diabetes, hypertension or other chronic diseases, abnormal child-bearing history, a history of drug use, and vitamin supplements—including vitamin D and calcium—that may affect metabolism or unavailable data. Pregnant women with singleton pregnancies and conceiving naturally were included. All pregnant women exerted a normal daily life without special sunlight exposure or quarantines. All study subjects were living in the Foshan, Guangdong Province of South China. Ethics approval was obtained by the Institutional Review Board (IRB) and Ethics Committee of Foshan Women and Children’s Hospital of Southern Medical University. All methods were carried out in accordance with relevant guidelines and regulations. Methods and laboratory testing followed standard operating procedures required for medical practice in Guangdong Province, including in this hospital. All participants provided written informed consent.

### Serum 25(OH)D analysis

Serum total 25(OH)D concentration was analyzed at gestational weeks 11 to 14 using a Mokosensor-A300 immune colloidal gold analyzer (MedicalSystems, Ningbo, China) following the manufacturer’s instructions. Briefly, 2 ml of blood was sampled from pregnant women at weeks 11–14 of gestation using an EDTA anti-coagulant blood tube. The blood sample was delivered to clinical laboratory immediately after sampling. Twenty microliters of whole blood were used for the analysis. The accuracy of the assay was from 85–115%, while the precision of the assay was ≤ 15% (coefficient of variation, CV). According to the Vitamin D Standardization-Certification Program (VDSCP) guidelines, a CV of < 15% is close to acceptable in the analysis [20–22].

The statuses of maternal serum 25(OH)D were categorized into three groups: 25(OH)D deficiency (< 50 nmol/L), 25(OH)D insufficiency (50–74 nmol/L), and 25(OH)D sufficiency (≥ 75 nmol/L).

### Chemistry analysis

Other blood chemistry parameters also came from the samples collected during weeks 11 to 14 of gestation. Specifically, alkaline phosphatase, pre-albumin, creatinine, cystatin C, calcium, and magnesium were analyzed on a Beckman-Coulter Automatic Chemistry Analyzer platform (Beckman-Coulter, Brea, CA, USA) following the manufacturer’s instructions and laboratory operating procedures. The collection of blood samples, including the selection of a collecting tube, followed routine clinical laboratory procedures.

### Glucose tolerance test

A 75 g oral glucose tolerance test (75 g OGTT) was performed between 24 and 28 weeks of gestation. Pregnant women who had a fasting glucose level < 5.1 before pregnancy were enrolled in the study. The diagnosis of GDM was defined according to the American Diabetes Association [23] using the following protocol: A 75 g oral glucose tolerance test (75 g OGTT) was performed between weeks 24 and 28 of gestation. Either of the following criteria met was diagnosed as GDM: fasting serum glucose ≥ 5.1 mmol/L, or 1 h of OGTT serum glucose ≥ 10 mmol/L, or 2 h of OGTT serum glucose ≥ 8.5 mmol/L).

### Coagulation test

The blood coagulation test was performed on a Stago STA-R Evolution (Diagnostica Stago, Asnières sur Seine Cedex, France) platform following the manufacturer’s instructions using a heparin anti-coagulant blood collection tube collected during weeks 11 to 14 of gestation. The coagulation parameters include thrombin time (TT), activated partial thromboplastin time (aPTT), fibrinogen (Fib), and D-dimer.

### Bone density examination

Bone density was measured using a Hong Yang BMD-1000 Ultrasound Bone Sonometer (Baoding, Hebei, China) by a specialist in our hospital following the protocol provided by the manufacturer. Pregnant women at weeks 11–14 of gestation were assigned for bone density measurement of the distal radius for 2 min. In brief, the Z value is obtained by comparing the sonographic value of the bone of a patient with a reference (same age, same sex). Thus, bone density was expressed as Z-scores.

### Statistical analysis

Statistical analysis was performed using the SPSS (version 24). Continuous variables are reported as mean ± standard deviation or median (interquartile), while categorical variables are presented as frequencies and percentages. For continuous variables, hypothesis testing for significant differences was performed using One-Way ANOVA and Nonparametric Tests for normal and non-normal distribution data, respectively. Pearson’s chi-square was used for categorical variables. Post Hoc Multiple Comparison was used to further identify significant differences among groups. Possible confounding factors with *p* < 0.20 were entered into multiple linear regression analysis to explore independent influencing factors of 25(OH)D. Spearman coefficient was used to determine the correlation between 25(OH)D status and pregnant outcomes. Unadjusted and adjusted logistic regression analysis were used to calculate the crude or adjusted odds ratios (OR), and their 95% confidence intervals (CI) were used to evaluate the relationship between 25(OH)D and GDM and other parameters (gestational adverse events). Variables with *p* < 0.10 in unadjusted analysis and possible confounding factors were entered into multivariable logistic regression procedure. The model’s adequacy was assessed by predicting correct percentage. A *p* < 0.05 were considered statistically significant.

## Results

### Association between maternal and clinical characteristics and 25(OH)D status

In this observation, the average 25(OH)D concentration was 59.1 ± 12.6 nmol/L; none of the women had 25(OH)D < 25 nmol/L. Four hundred and thirty-four (434, 28.6%) women had 25(OH)D < 50 nmol/L, 882 women (58.2%) had 25(OH)D insufficiency (50–74 nmol/L), and 200 (13.2%) women had 25(OH)D ≥ 75 nmol/L. Pre-adjusted analysis using the Chi-square test, One-way ANOVA, and nonparametric test results that the maternal and clinical characteristics are listed in Table 1 on the basis of 25(OH)D status. Table 1 also shows that serum 25(OH)D varies significantly in regard to season at sampling. Serum alkaline phosphatase (ALP), pre-albumin, creatinine, and thrombin time (TT) also differed upon different serum 25(OH)D status (p < 0.05 for all). However, the categories of pre-albumin and creatinine, as well as the rest of variables in the list, do not show difference based on 25(OH)D status.

**Table 1:** Comparison of general factors among groups based on vitamin D status (Chi-square test, One-way ANOVA, and nonparametric test)

Parameters	n	25(OH)D deficient (< 50 nmol/L)(*n* = 434)	25(OH)D insufficient (50–74 nmol/L) (*n* = 882)	25(OH)D sufficient (≥ 75 nmol/L) (*n* = 200)	*P* value
Age (year)	1516	29.57 ± 4.72	29.35 ± 4.55	29.35 ± 4.43	0.696
Intrapartum BMI (kg/m^2^)	1516	26.77 ± 3.23	26.81 ± 3.31	26.54 ± 3.05	0.574
Parity	1510				0.073
0	784	221 (50.9)	457 (51.8)	106 (53.0)	
1	652	196 (45.2)	380 (43.1)	76 (38.0)	
≥ 2	80	17 (3.9)	45 (5.1)	17 (9.0)	
Season at sampling	1516				< 0.001
Spring	547	257 (59.2)	255 (28.9)	35 (17.5)	
Summer	575	128 (29.5)	365 (41.4)	82 (41.0)	
Autumn	248	1 (0.2)	176 (20.0)	71 (35.5)	
Winter	146	48 (11.1)	86 (9.8)	12 (6.0)	
Gestational weeks at sampling	1516				0.181
11	174	59 (13.6)	87 (9.9)	28 (14.0)	
12	798	228 (52.5)	460 (52.2)	110 (55.0)	
13	373	102 (23.5)	232 (26.3)	39 (19.5)	
14	171	45 (10.4)	103 (11.7)	23 (11.5)	
Bone function					
Bone density Z value	924	-0.54(0.68)	-0.54(0.62)	-0.59(0.66)	0.667
Ca^2+^ (mmol/L)	1478	1.37 ± 0.13	1.38 ± 0.13	1.38 ± 0.12	0.418
Mg^2+^ (mmol/L)	1477	1.33 ± 0.15	1.33 ± 0.15	1.31 ± 0.13	0.137
ALP (IU/L)					0.008
< 45	63	27 (10.2)	30 (5.2)	6 (4.5)	
45–125	822	221 (83.7)	482 (84.0)	119 (88.8)	
≥ 125	87	16 (6.1)	62 (10.8)	9 (6.7)	
Liver and kidney function					
Pre-ALB (mg/L)	951	216.74 ± 30.95	224.64 ± 33.28	226.94 ± 33.26	0.002
Pre-ALB (mg/L)					0.769
< 150	12	2 (0.8)	8 (1.4)	2 (1.5)	
≥ 150	939	255 (99.2)	552 (98.6)	132 (98.5)	
Cre (µmol/L)	1466	47.84 ± 7.01	46.78 ± 7.11	47.16 ± 7.99	0.047
Cre (µmol/L)					0.550
< 41	234	60 (14.3)	141 (16.5)	33 (16.9)	
≥ 41	1232	359 (85.7)	711 (83.5)	162 (83.1)	
Cys C (mg/L)	323	0.97 ± 0.22	0.96 ± 0.21	0.94 ± 0.26	0.690
Coagulation function					
D-Dimer (mg/L)	1431	0.37(0.24)	0.39(0.23)	0.38(0.23)	0.232
Fibrinogen (g/L)	1437	3.98 ± 0.634	4.03 ± 0.67	4.05 ± 0.66	0.385
aPTT (s)	1437	36.28 ± 3.03	36.24 ± 2.86	36.65 ± 3.03	0.201
TT (s)					< 0.001
< 14	192	28 (6.9)	123 (14.7)	41 (20.9)	
≥ 41	1245	379 (93.1)	711 (85.3)	155 (79.1)	
PLT (*10^9/L)	1497	252.13 ± 54.48	250.32 ± 54.69	246.78 ± 53.46	0.521

*BMI* Body mass index, *Ca2* + calcium, *Mg2* + magnesium, *ALP* Alkaline phosphatase, *ALT* Alanine aminotransferase, *Pre-ALB* pre-albumin, *Cre* creatinine, *Cys C* cystatin C, *aPTT* activated partial thromboplastin time, *TT* Thrombin time, *PLT* Platelet

### Association between parity, season at sampling, and laboratory parameters and 25(OH)D concentrations

Tables 2 and 3 show the association between maternal serum 25(OH)D concentrations and parity, season at sampling, serum alkaline phosphatase. Women going into birth ≥ 2 times had a higher 25(OH)D concentration than those with 1 or 0 paritys (*p* = 0.018 and *p* = 0.044 respectively). The same result was found in adjusted linear analysis (*p* = 0.010 and *p* = 0.032, respectively). There was a significant difference in 25(OH)D concentrations among samples collected in different seasons; the 25(OH)D concentrations assended in the order of spring, winter, summer, and autumn. There was a significant difference between serum 25(OH)D concentration and alkaline phosphatase activities, of which a higher serum 25(OH)D concentration tended to have higher alkaline phosphatase activities (*p* = 0.005 and 0.004, respectively).

**Table 2:** Stratification analysis of the correlation between parity, season at sampling, alkaline phosphatase, and 25(OH)D concentrations (One-way ANOVA, Post Hoc Multiple Comparison)

Parameters	25(OH)D concentration	Group A	Group B	*P* value	95% CI
Parity					
0	59.2 ± 12.6	0	1	0.415	(-0.76, 1.85)
1	58.7 ± 12.4		≥ 2	0.044	(-5.85, -0.08)
≥ 2	62.2 ± 13.1	1	≥ 2	0.018	(-6.42, -0.59)
Season at sampling					
Spring	53.7 ± 11.2	Spring	Summer	< 0.001	(-8.43, -4.73)
Summer	60.3 ± 12.3		Autumn	< 0.001	(-18.08, -14.20)
Autumn	69.8 ± 8.8		Winter	0.021	(-5.94, -0.32)
Winter	56.8 ± 11.4	Summer	Autumn	< 0.001	(-11.56, -7.57)
			Winter	0.009	(0.60, 6.30)
		Autumn	Winter	< 0.001	(-15.92, -10.10)
ALP					
< 45	54.8 ± 13.2	< 45	45–125	0.005	(-7.90, -1.41)
45–125	59.5 ± 12.7		> 125	0.004	(-10.19, -1.98)
≥ 125	60.9 ± 11.6	45–125	> 125	0.316	(-4.23, 1.37)

**Table 3:** Association between parity and serum 25(OH)D concentration (multiple linear regression)

Parameters	25(OH)D		
	Mean ± standard	B (95%CI)	*P* value
Parity			
0	59.2 ± 12.6	-2.841 (-5.443, -0.240)	0.032
1	58.7 ± 12.4	-3.439 (-6.066, -0.813)	0.010
≥ 2	62.2 ± 13.1	1 (reference)	
Season at sampling			
Spring	53.7 ± 11.2	-3.123 (-5.195, -1.052)	0.003
Summer	60.3 ± 12.3	3.485 (1.424, 5.545)	0.001
Autumn	69.8 ± 8.8	13.055 (10.742, 15.368)	< 0.001
Winter	56.8 ± 11.4	1 (reference)	
Gestational weeks at sampling			
11	59.5 ± 13.8	1.175 (-1.213, 3.563)	0.335
12	59.2 ± 12.6	0.993 (-0.881, 2.868)	0.299
13	58.9 ± 12.1	0.499 (-1.551, 2.548)	0.633
14	59.0 ± 12.4	1 (reference)	

### Correlation of 25(OH)D status with GDM and other pregnancy adverse events

As shown in Table 4, results from spearman correlation analysis indicate that there is no correlation between vitamin D status and adverse outcomes in early gestating women (*p* > 0.05 for all). Interestingly, the rate of 1 h GTT positive (*r* = -0.037) and 2 h GTT positive (*r* = -0.037) was inversely correlated with the increament of serum 25(OH)D status, but with no significance (*p* = 0.145 and 0.534, respectively).

**Table 4:** Correlation between vitamin D status and gestational outcomes

Clinical outcomes	n	Vitamin D deficient (< 50 nmol/L) (*n* = 434)	Vitamin D insufficient (50–74 nmol/L) (*n* = 882)	Vitamin D sufficient (≥ 75 nmol/L) (*n* = 200)	r	*P* value
Fasting glucose	1516				0.028	0.279
< 5.1	1478	427 (98.4)	856 (97.1)	195 (97.5)		
≥ 5.1	38	7 (1.6)	26 (2.9)	5 (2.5)		
Glucose of 1 h GTT	1516				-0.037	0.155
< 10	1358	382 (88.0)	793 (89.9)	183 (91.5)		
≥ 10	158	52 (12.0)	89 (10.1)	17 (8.5)		
Glucose of 2 h GTT	1516				-0.037	0.145
< 8.5	1342	376 (86.6)	786 (89.1)	180 (90.0)		
≥ 8.5	174	58 (13.4)	96 (10.9)	20 (10.0)		
GDM	1516				-0.016	0.534
No	1252	357 (82.3)	725 (82.2)	170 (85.0)		
Yes	264	77 (17.7)	157 (17.8)	30 (15.0)		
GH	1516				0.030	0.236
No	1492	429 (98.8)	868 (98.4)	195 (97.5)		
Yes	24	5 (1.2)	14 (1.6)	5 (2.5)		
PROM	1516				-0.047	0.068
No	1240	345 (79.5)	724 (82.1)	171 (85.5)		
Yes	276	89 (20.5)	158 (17.9)	29 (14.5)		
Fetal distress	1516				-0.031	0.235
No	1400	393 (90.6)	823 (93.3)	184 (92.0)		
Yes	116	41 (9.4)	59 (6.7)	16 (8.0)		
Preterm	1516				-0.033	0.202
No	1432	405 (93.3)	836 (94.8)	191 (95.5)		
Yes	84	29 (6.7)	46 (5.2)	9 (4.5)		
PPH	1516				0.028	0.268
No	1439	416 (95.9)	835 (94.7)	188 (94.0)		
Yes	77	18 (4.1)	47 (5.3)	12 (6.0)		
LBW	1516				-0.025	0.325
No	1440	409 (94.2)	839 (95.1)	192 (96.0)		
Yes	76	25 (5.8)	43 (4.9)	8 (4.0)		
Delivery mode					0.045	0.087
Vaginal	840	231 (54.0)	489 (57.2)	120 (61.2)		
Cesarean	639	197 (46.0)	366 (42.8)	76 (38.8)		

*GDM* gestational diabetes mellitus, *GH* gestational hypertension, *PROM* premature rupture of membrane, *PPH* postpartum hemorrhage, *LBW* low body weight

### Association between maternal 25(OH)D status and gestational diabetes mellitus

Logistic regression analysis was performed to assess the association between serum 25(OH)D and gestational diabetes mellitus. In this study, we found that there was no association between 25(OH)D and the risk of GDM, as shown in Table 5. In unadjusted logistic regression analysis, no significant difference was observed between maternal 25(OH)D and GDM (*p* = 0.627). After adjusted by age, parity, and season at sampling, women with serum 25(OH)D ≥ 75 nmol/L (vitamin D sufficient) were grouped as the reference group, while women whose serum 25(OH)D between 50–74 nmol/L (vitamin D insufficient), or < 50 nmol/L (vitamin D defficient) groups were compared with the reference group. The results indicated that there was no association between serum 25(OH)D and GDM, with an odds ratio of 1.250 (95%CI 0.763–2.046, *P* = 0.376) and 1.224 (95%CI 0.794–1.888, *P* = 0.360), respectively. GDM women were more likely to be older than those without GDM.

**Table 5:** Association between 25(OH)D status and gestational diabetes mellitus

Parameters	Non-GDM	GDM	*P* value	Multivariable-adjusted	
				OR (95%)	*P* value
Age			< 0.001		
< 35	1098 (84.1)	207 (15.9)		1 (reference)	
≥ 35	154 (73.0)	57 (27.0)		1.086 (1.266–2.576)	0.001
Parity			0.107		
0	662 (84.4)	122 (15.6)		1 (reference)	
1	528 (81.0)	124 (19.0)		1.142 (0.857–1.523)	0.364
≥ 2	62 (77.5)	18 (22.5)		1.367 (0.769–2.430)	0.287
25(OH)D			0.627		
≥ 75 nmol/L	170 (85.0)	30 (15.0)		1 (reference)	
50–74 nmol/L	725 (82.2)	157 (17.8)		1.250 (0.763–2.046)	0.376
< 50 nmol/L	357 (82.3)	77 (17.7)		1.224 (0.794–1.888)	0.360
Season at sampling			0.334		
Spring	459 (83.9)	88 (16.1)		1 (reference)	
Summer	475 (82.6)	100 (17.4)		1.098 (0.793–1.521)	0.573
Fall	205 (82.7)	43 (17.3)		1.137 (0.737–1.755)	0.561
Winter	205 (82.7)	33 (22.6)		1.472 (0.932–2.325)	0.097

## Discussion

The aim of this study was to assess vitamin D status in pregnant women in the Foshan area of Guangdong, China and to test for an association between gestational diabetes and adverse pregnancy events, and to compare those results with other reports [24–30].

Our study results did not show an association between maternal serum 25(OH)D status and GDM and other types of pregnant adverse events, including gestational hypertension, premature rupture of membrane, postpartum hemorrhage, and low body weight of newborns. This result is similar with a previous meta analysis report [31]. A randomized controlled trial also showed a limitation of vitamin D supplementation for GDM prevention in vitamin D sufficient populations [32].

Our results showed that the mean 25(OH)D concentration of the subjects was 59.1 nmol/L. Comparing to other studies in China, our study indicated that serum 25(OH)D < 50 nmol/L in early pregnant women was a relatively low prevalence (28.6%). While another report showed that the mean serum 25(OH)D in pregnant women was 40.1 nmol/L and 25(OH)D < 50 nmol/L accounted for 76.4% in Hefei [33]; a study conducted by Song et al. [34] also reported that the mean maternal 25(OH)D concentration was 28.6 nmol/L and more than 90% of pregnant women had 25(OH)D < 50 nmol/L in Beijing. These contradictory results might be attributed to the analytical methods used in different studies. For instance, serum 25(OH)D analysis can be measured by LC–MS/MS spectrometry, chemiluminescent immunoassay, high performance chromatography (HPLC), and immune colloidal gold methods, etc. This phenomenon could be due to the climate and sun exposure difference between the South and the North. Our results showed that sampling at different seasons had an effect on serum 25(OH)D concentrations, in accordance with public understanding. There was also a correlation between serum alkaline phosphatase, pre-albumin, creatinine, and thrombin time and serum 25(OH)D concentrations.

Since the significance of association between parity and serum 25(OH)D concentration before adjustment was weak, we performed additional stratified analyses using One-Way ANOVA and Post Hoc Multiple Comparison, followed by multiple linear regression. Results indicated a positive association between maternal serum 25(OH)D concentration and the number of parity. One study mentioned that there was no significant difference between serum 25(OH)D concentration and age, parity, or gestational age [35], and no association between parity and 25(OH)D concentration was observed in another study [36]. While Shen et al. found that high parity was associated with increasing serum vitamin D status [37]. The mechanism underlying this phenomenon is therefore unclear. There could be certain compensation in metabolism function during pregnancy that requires further investigation. From our clinical experience in Foshan China, women gained experience in how to deal with pregnancy with more parity going; they tended to get more sun exposure and a nutrient-rich diet, and these facts could have contributed to the results.

Women who had high serum vitamin D also had higher alkaline phosphatase activity. A study observed that there was an association between alkaline phosphatase activity and moderate vitamin D deficiency group; however, the correlation was not significant [38]. Another study also found that there was no correlation between alkaline phosphatase activity and vitamin D status in school children [39].

Serum alkaline phosphatase activity is widely used in clinical practice as a marker of bone turnover [40]. Medical conditions with osteolysis had elevated serum alkaline phosphatase activity levels [41]. Based on our results, we hypothesize that there is a relationship between vitamin D and alkaline phosphatase in maintaining bone and mineral metabolic homeostasis—a hypothesis that merits further investigation.

Serum pre-albumin could be a marker indicating malnutrition and other potential pathological conditions, such as inflammation, in clinical practice [42]. In interpreting pregnant women who had low serum vitamin D status (vitamin D deficient) and low pre-albumin concentration, we hypothesize that there is a relationship between serum vitamin D and pre-albumin, either acting as a nutrition factor or inflammation index, as also suggested in other study [43]. The detailed mechanism of the association between these two factors requires further investigation. Liu et al. [44] found that serum pre-albumin levels were correlated with gestational diabetes mellitus status, and they hypothesized that pre-albumin can be used as an indicator to reflect the presence of gestational diabetes mellitus.

Serum creatinine is considered a useful biomarker for diagnosis of renal function and is widely used in clinical practice [45, 46]. One study found that vitamin D receptor activation increased creatinine generation and serum creatinine level and may play a role in renal function regulation [47]. Higher serum creatinine levels in vitamin D deficient pregnant women may imply the activation of the vitamin D receptor occurred as a compensatory effect and merits further investigation. Saibene et al. found that 25-hydroxy vitamin D insufficiency in total thyroidectomy patients was associated with lower serum creatinine, which indicated a possible role of creatinine in the risk of transient postoperative hypocalcemia and vitamin D metablic pathway [48].

Per the positive correlation between serum 25(OH)D concentration and thrombin time (TT) in pregnant women, our study implies that there is an association between 25(OH)D and blood coagulation. A previous study showed that treatment with vitamin D supplementation had a prothrombotic effect in patients with vitamin D insufficiency, an issue requiring further study [49].

## Conclusions

This study did not show an association between maternal serum vitamin D status and GDM. There was a positive correlation between serum vitamin D and parity, serum alkaline phosphatase, pre-albumin levels, and a negative correlation between serum vitamin D and thrombin time and serum creatinine levels, which could have potential clinical significance and requires further investigation.

### Limitations of this study

Since serum 25(OH)D concentrations is affected by several factors, such as seasons, geographical location, race, diet/lifestyle, medical conditions, etc., it is important to develop a more rigourous protocol when performing future vitamin D related studies. In this study, patients enrollment, diet and sun exposure, maternal nutrition supplement, etc. could be more accurately controlled. The analyzer used in 25(OH)D assay could be improved if the budget allowed, such as an LC–MS/MS spectrometer. Otherwise, more accurate methods and standardization of the assay should be performed in a future study and in clinical testing. For nstance, we would re-measure 100–150 samples based on a VDSCP assay along with standard reference materials (SRM) and applying Deming regression to standardize their values in the full sample. Socioeconomic status, lifestyle, the eating habits of pregnant woman, the monitoring and classification of gestational weight gain of the pregnancy women, and their exercise levels are factors of limitation in this study and should be considered in the future study. The study population mainly composed of Han Chinese women, which ensured data homogeneity, but it may under-represent population groups with different lifestyles and socioecomic situations, leading to biased results.

## Data Availability

Data is available from the corresponding author upon inquiry.
